# Insertion Guidance Based on Impedance Measurements of a Cochlear Electrode Array

**DOI:** 10.3389/fncom.2022.862126

**Published:** 2022-06-23

**Authors:** Enver Salkim, Majid Zamani, Dai Jiang, Shakeel R. Saeed, Andreas Demosthenous

**Affiliations:** ^1^Department of Electronic and Electrical Engineering, University College London (UCL), London, United Kingdom; ^2^Department of Electronic and Electrical Engineering, Biomedical Device Technology Group, Muş Alparslan University, Muş, Turkey; ^3^UCL Ear Institute, London, United Kingdom

**Keywords:** cochlear implant, computational models, electrode proximity, impedance variation, parameterization

## Abstract

The cochlear implantable neuromodulator provides substantial auditory perception to those with severe or profound impaired hearing. Correct electrode array positioning in the cochlea is one of the important factors for quality hearing, and misplacement may lead to additional injury to the cochlea. Visual inspection of the progress of electrode insertion is limited and mainly relies on the surgeon's tactile skills, and there is a need to detect in real-time the electrode array position in the cochlea during insertion. The available clinical measurement presently provides very limited information. Impedance measurement may be used to assist with the insertion of the electrode array. Using computational modeling of the cochlea, and its local tissue layers merging with the associated neuromodulator electrode array parameters, the impedance variations at different insertion depths and the proximities to the cochlea walls have been analyzed. In this study, an anatomical computational model of the temporal region of a patient is used to derive the relationship between impedance variations and the electrode proximity to the cochlea wall and electrode insertion depth. The aim was to examine whether the use of electrode impedance variations can be an effective marker of electrode proximity and electrode insertion depth. The proposed anatomical model simulates the quasi-static electrode impedance variations at different selected points but at considerable computation cost. A much less computationally intensive geometric model (~1/30) provided comparative impedance measurements with differences of <2%. Both use finite element analysis over the entire cross-section area of the scala tympani. It is shown that the magnitude of the impedance varies with both electrode insertion depth and electrode proximity to the adjacent anatomical layers (e.g., cochlea wall). In particular, there is a 1,400% increase when the electrode array is moved very close to the cochlea wall. This may help the surgeon to find the optimal electrode position within the scala tympani by observation of such impedance characteristics. The misplacement of the electrode array within the scala tympani may be eliminated by using the impedance variation metric during electrode array insertion if the results are validated with an experimental study.

## 1. Introduction

The cochlea has a vital role in generating a sense of hearing. It transforms the sound waves into mechanical vibrations of the hair cells and subsequently into electrical pulses. The pulses are transmitted to the brain through the auditory nerve to provide hearing sensation. Sensorineural hearing loss is caused by damage to the inner ear, especially the hair cells, or the dysfunction of the auditory nerve (Svirsky et al., 2004). This is a socioeconomic burden and has led to substantial constraints globally. Over 5% of the world's population suffers from hearing loss (432 million adults and 34 million children) (Kushalnagar, 2019). The available solutions are varied depending on the type of hearing loss and include hearing aids, cochlear implants (CIs), and other assistive devices (Kushalnagar, 2019). The CI is a neural prosthesis designed to restore hearing loss by electrical stimulation of the auditory nerve. Using an electrode array inserted in the scala tympani of the cochlea, the implant can deliver modulated electric stimuli directly to the residual auditory nerve fibers, thus replacing the function of the damaged hair cells (Dang, 2017; Dhanasingh and Jolly, 2017).

Over the decades, conventional surgery using CIs remains essentially unchanged and is generally considered safe and effective (Caversaccio et al., 2019). Although advancements in CI design have been reported (Hajioff, 2016; Dazert et al., 2017; Dhanasingh and Jolly, 2017), the quality of restored hearing sensation is strongly related to the quality of the CI surgery, the design of the electrode structure, and the insertion tools and techniques (Tan et al., 2013). As the electrode array is inserted mainly guided by touch, it has been reported that partial insertion, deformation of the electrode array, and even penetration of the basilar membrane can occur which prejudices the performance of hearing after implantation (Rebscher et al., 2008). Obtaining the optimum positioning of the electrode array during cochlear electrode implantation is essential for the preservation of residual hearing and improved clinical outcomes (Finley and Skinner, 2008). Misplacement of the electrode array may lead to further hearing loss and insertion trauma if the electrode array touches the cochlea wall (Holden et al., 2013; Min et al., 2013). Furthermore, if the electrode array touches sensitive layers of the cochlea such as a basilar membrane or osseous spiral lamina layers due to significant variability in the size of the human scala tympani (Skinner et al., 2002; O'Connell et al., 2016), it may lead to severe trauma. Also, it has been shown that there is a significant correlation between hearing outcomes and the correct placement of electrode arrays entirely in the scala tympani (Wanna et al., 2014). It is, therefore, important that the electrode array should be positioned accurately within the scala tympani to minimize such consequences and improve hearing outcomes (O'Connell et al., 2016).

There are some emerging concepts, such as careful surgical techniques and training, new designs of the electrode structure, and novel insertion tools (electrode arrays with softer material, pre-curved perimodiolar arrays, and Advance Off-Stylet insertion technique are some examples), that may help reduce insertion mishaps and intracochlear trauma. Surgeons have no real-time feedback about electrode status while inserting the electrode array into the cochlea (Jethanamest et al., 2010; Tan et al., 2013). It has been shown that a magnetically guided system (Clark et al., 2012) and robotic insertion can help control insertion forces by varying insertion speed (Zhang et al., 2010). These systems may reduce trauma, but real-time local position information of the electrode array in the cochlea during insertion is required. The electrode position can be monitored using medical imaging (e.g., computer tomography). While this method may help to accurately place the electrodes, it is not suitable due to the danger of radiation risk on the patient, and it is rarely done intra-operatively (Giardina et al., 2017). Alternatively, the electrode array position can be rapidly assessed from the implant at the time of implantation by electrically-evoked neural responses, electric field imaging, or impedance variations (Mens, 2007). The first method may not be reliable due to the highly variable results reported (Miller et al., 2008; Mittmann et al., 2015). Although the major error position of the electrodes in the scala tympani could be registered using electric field imaging, it was not utilized to predict the positions of the electrodes in the scala tympani (Vanpoucke et al., 2011).

Using impedance measurements can be a safer and more reliable method to help determine the relation of the electrodes' position to the cochlea wall during surgery (Mens, 2007; Tan et al., 2013; Newbold et al., 2014). It has been shown that perilymph (fluid in the scala tympani) has relatively higher conductivity than bone and cochlea wall, leading to the hypothesis that the measured electrode impedance to ground should be higher when an electrode approaches the cochlea wall compared to when the electrode is in the middle of the scala (Frijns et al., 1995). Thus, impedance measurement can be an option to monitor the proximity of the electrode array to the cochlea wall in real-time to prevent any damage, and find the optimum position for the electrode during the insertion process (Mens, 2007; Tan et al., 2013; Newbold et al., 2014; Giardina et al., 2017).

**Figure 1:**
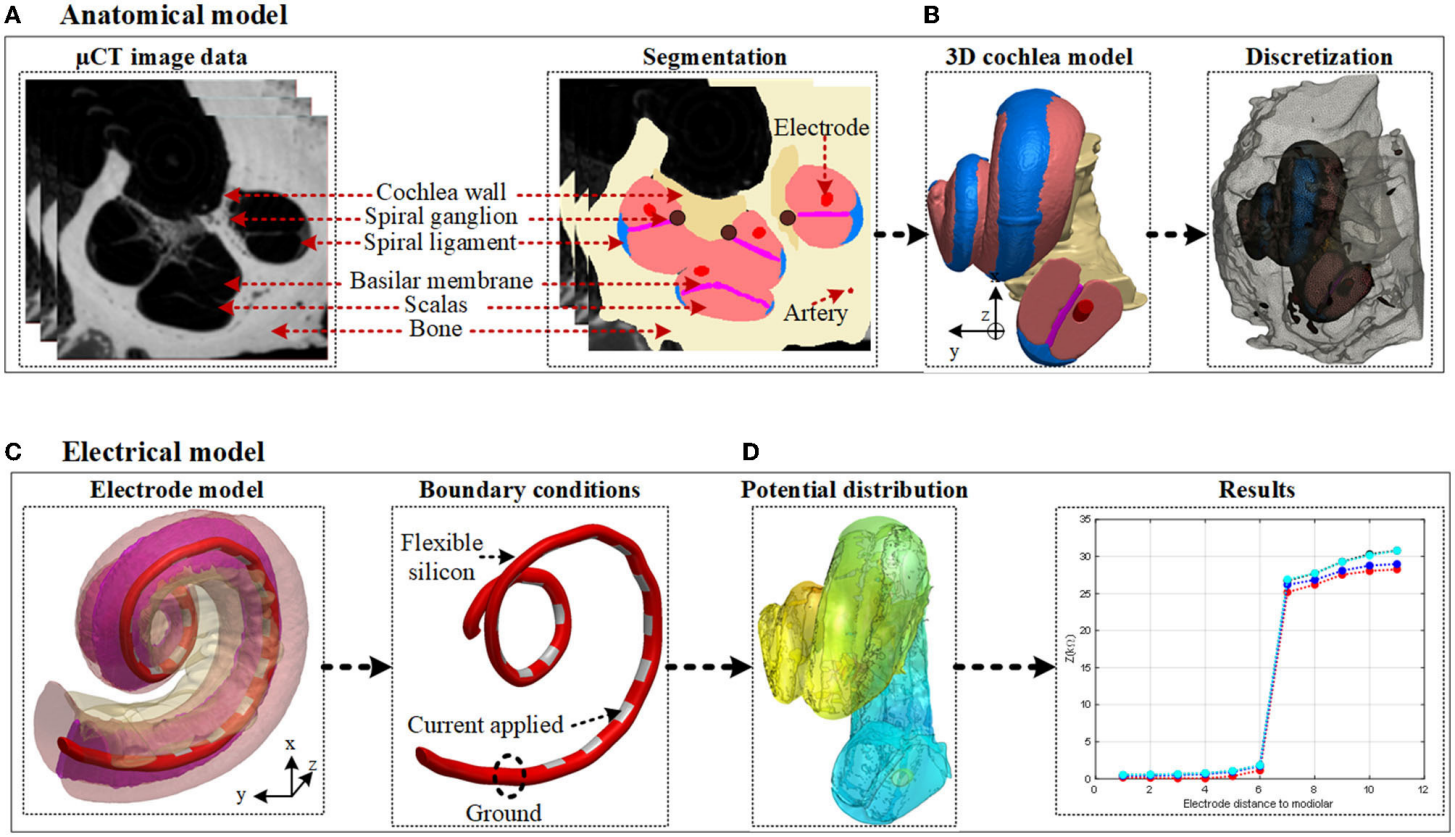
Human cochlea modeling and electrical potential simulation. **(A)** The anatomical layers are segmented based on the image dataset. **(B)** 3D finite element method (FEM) domains are constructed based on captured individual image sets using associated labeling and smoothing filters in ScanIP software. **(C)** The electrode model is generated and embedded in the constructed 3D cochlea. The model is then discretized, and a current source is applied using appropriate boundary conditions. **(D)** The electrical potential distribution in the volume conductor is resolved by FEM and the impedance variation is calculated.

As the human cochlea is embedded deep inside the temporal bone and there is geometrical variation in the size of the scala tympani, direct measurements of electrical potential or impedance may not be readily feasible (Bai et al., 2019). Also, using conventional techniques it may not be feasible to conduct systematic comparison across individuals to examine the precise position of the electrode (Pile et al., 2017). Computational cochlea models have been utilized to simulate the current spread in the cochlea and provided useful insights (Malherbe et al., 2016; Salkim et al., 2020). Such models are implemented using the finite element method (FEM). The models consist of a volume conductor that accounts for various anatomical structures and the inserted electrode array by their respective conductivities and appropriate boundary conditions. This study examines the relationship between electrode impedances to the ground and their proximity to adjacent layers, and their insertion depth using accurate FEM computation models. A multi-layered anatomical three-dimensional (3D) volume conductor model of the human cochlea was generated using micro-CT (μCT) datasets as shown in Figures 1A,B. An electrode array was generated based on the Advanced Bionics HiFocus^*TM*^ SlimJ electrode (Hannover, Germany) and combined with the anatomical volume conductor of the cochlea as shown in Figure 1C. Twelve different models were generated in X and Y proximity and insertion depth in the z direction used for all electrode insertions. The models were simulated, and the results were analyzed (as shown in Figure 1D) to examine if the impedance variation can be used as a marker for electrode position guidance.

The computation cost using the anatomical cochlea model limits the quantity of information that can be examined. More detailed information about different electrode proximities can be investigated using an adequately accurate and simpler geometric model at a much lower computation cost. A 3D geometrical FEM model was generated by imitating the anatomical model of the cochlea and neuromodulator parameters to readily parameterize the electrode array proximity to the cochlea (shown in Figure 4). The impact of the different proximities and insertion depths of the electrodes in the scala tympani was evaluated using impedance distribution analysis to determine whether the safe position of the electrode array could be predicted from impedance measurements. The electrode array proximity was parameterized in x and y directions for each insertion depth in the z direction. Different models were generated by selecting samples in X and Y positions. This resulted in 144 different electrode proximity models for 16 different electrode insertion combinations. The impedance variation was simulated and recorded for all significant electrode array proximities in the scala tympani using the geometrical FEM model. Useful information was obtained using a multi-layered anatomical model but at high computation cost and time. Using a geometrical cochlea model enabled multiple detailed measurements of impedance variation vs. proximity of the electrode array to the adjacent layers at reasonable computation cost and time. The results showed that the magnitude of the impedance significantly varied with both electrode insertion depth and proximity to the cochlea wall.

The rest of the article is organized as follows. Section 2 describes methods to generate anatomical and geometric volume conductors of the cochlea, electrode array design, and quasi-static electrical potential simulation. The results of electrode proximity to the anatomical 3D cochlea wall and insertion depth based on impedance variation are presented in Section 3. Discussion on the results is reported in Section 4, and conclusions in Section 5.

## 2. Methods

For all simulations, a computer with an Intel Core i7-6700 CPU at 3.4 GHz with 64 GB RAM was used.

### 2.1. Cochlea Anatomical Model Development

The process of image data segmentation involves the construction of the cochlea volume conductor and its associated anatomical layers. These include the scalas, cochlea wall, basilar membrane, spiral ganglion, spiral ligament, artery, and bone.

#### 2.1.1. Micro-CT Data and Segmentation Process

To obtain accurate FEM results, it is important to develop a 3D anatomical model of the inner ear within the cochlea. The volume conductor of the cochlea and the layers in its vicinity were generated based on a high-resolution (2.24 × 2.24 × 5 μm) voxel size μCT image stack of a human cochlea (Avci et al., 2014) as shown in Figure 1A. Due to limited computation memory, the effective operative field of the scans was rescaled to include only the cochlea and its immediate surroundings and was later down-sampled to an isotropic resolution of 9.6 μm with a spatial resolution of 930 × 930 × 1,014 voxels.

The μCT data was imported to Simpleware ScanIP v2016.09 (Synopsys, Mountain View, USA) for image processing and data segmentation by defining regions in the image data that belong to the same anatomical layers. In this way, it becomes possible to construct 3D models that represent the anatomical layers. The detailed cochlea volume conductor was composed of the scala tympani, scala vestibuli, cochlea wall, basilar membrane, spiral ligament, stria vascularis, spiral ganglion, and associated arteries as listed in Table 1. The outermost layer that surrounds the cochlea was designated as the bony layer. Both automatic and manual segmentation processes were used to obtain a highly efficient and reliable model for simulation (Salkim et al., 2019). Smoothing filters utilizing recursive Gaussian, median, and mean filters were employed to allocate each tissue layer in a specific grayscale range. Each tissue layer was then generated based on an automatic segmentation process using this grayscale. Manual segmentation was used when editing the morphology or filling cavities (i.e., dilate, erode, open, and close functions) were used in ScanIP software. To obtain appropriate boundaries and remove any overlapping sections between the tissue layers, Boolean operations were applied.

**Table 1:** Tissue conductivities of cochlea structures used in finite element method (FEM) models of the cochlea.

**Tissue layer**	**Conductivity (S/m)**	**References**
Scalas	1.43	Finley et al., 1990
Cochlea wall	0.3	Finley et al., 1990
Basilar membrane	0.0125	Hanekom and Hanekom, 2016
Spiral ligament	1.67	Frijns et al., 1995
Stria vascularis	0.0053	Frijns et al., 1995
Spiral ganglion	0.33	Hanekom and Hanekom, 2016
Artery	0.32	Gabriel et al., 1996
Bone	0.0156	Finley et al., 1990; Hanekom and Hanekom, 2016
Silicone	1e-7	Hanekom and Hanekom, 2016

#### 2.1.2. Generation of a 3D Model of the Cochlea

After labeling all tissue layers and their edges on image data, the 3D model of each tissue layer in the cochlea was generated as shown in Figure 1B to enable simulation of the electrical potential distribution generated by a given electrode setting. The added computation time due to sharp edges were reduced by applying 3D editing filters in ScanIP software. Spiral ganglion and nerves are distributed throughout the tunnel spiral in the modiolus called the Rosenthal's canal. Since they possess similar conductivities, they were considered as one layer for potential distribution analysis. The basilar membrane and the osseous spiral lamina layers were combined and modeled as one layer due to the discontinuity of the osseous spiral lamina. The thin membranes between the scala vestibuli and the vestibule were excluded when modeling as they cannot be identified in the μCT scans. Since the stria vascularis layer is comparatively thin, it was modeled for all models as “contact impedance” during simulations. Practical computation times were attained using these adjustments.

### 2.2. Electrode Array Design in the Anatomical Model

To conduct stimulation currents to different parts of the cochlea, an electrode array model was based on a commercially available electrode [Advanced Bionics HiFocus^*TM*^ SlimJ electrode (Hannover, Germany)] with 16 platinum electrodes. The electrodes provide adequate quality of cochlea stimulation (Dhanasingh and Jolly, 2017). The 16 platinum electrodes are supported by flexible silicone and are designed to face the inner cochlea wall (Figure 1C).

**Figure 2:**
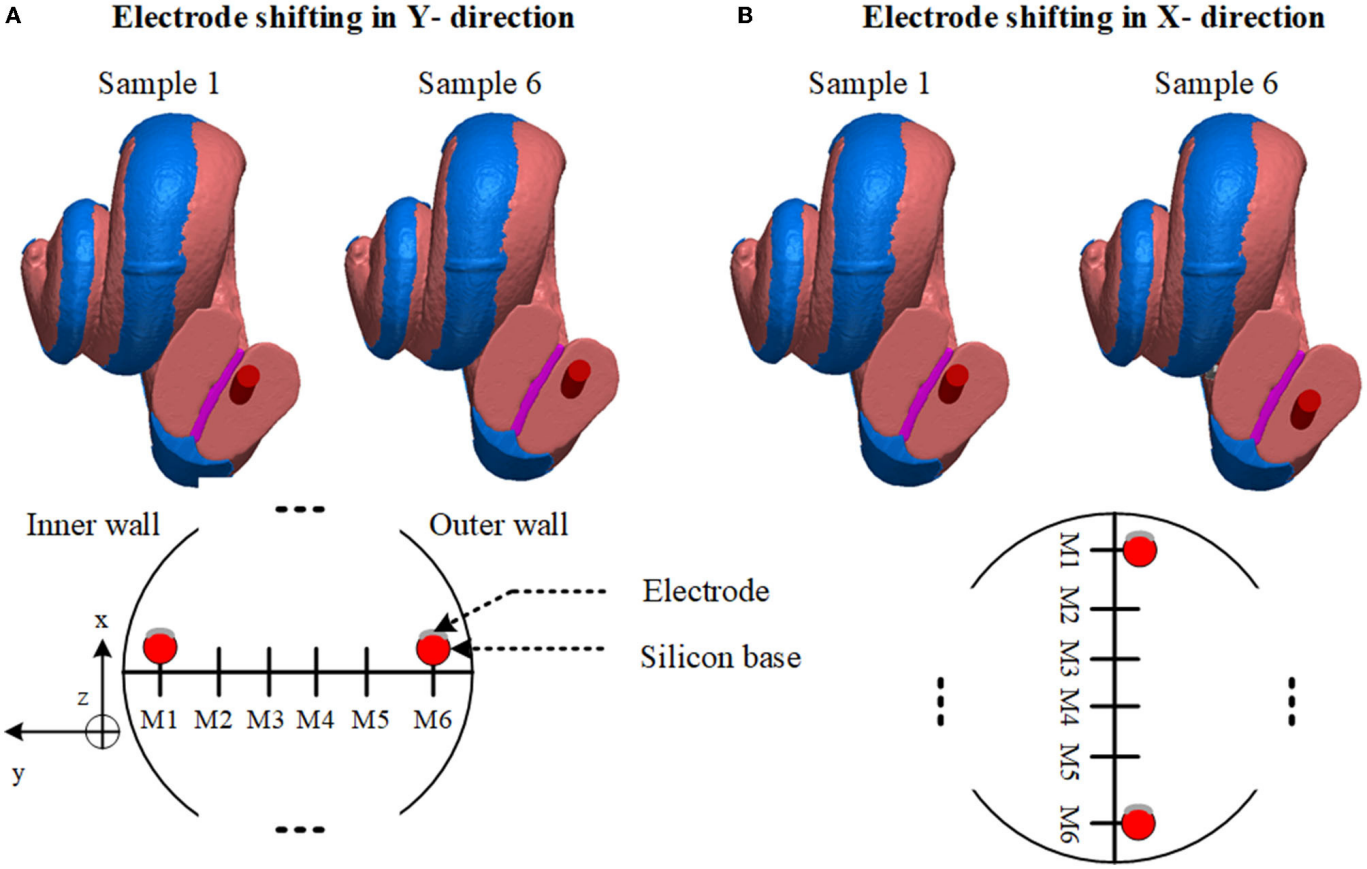
Anatomical layers and electrode array positions. **(A)** Sample 1 shows the electrode array nearly touching the basilar membrane. Sample 6 shows near the outer wall. Lower: diagram of electrode sweeps in the in-y direction. **(B)** Sample 1 shows a relatively closer model to the cochlea wall. Sample 6 shows near the lower wall. Lower: Diagram of electrode sweep in the x direction. Models are equally spaced.

**Figure 3:**
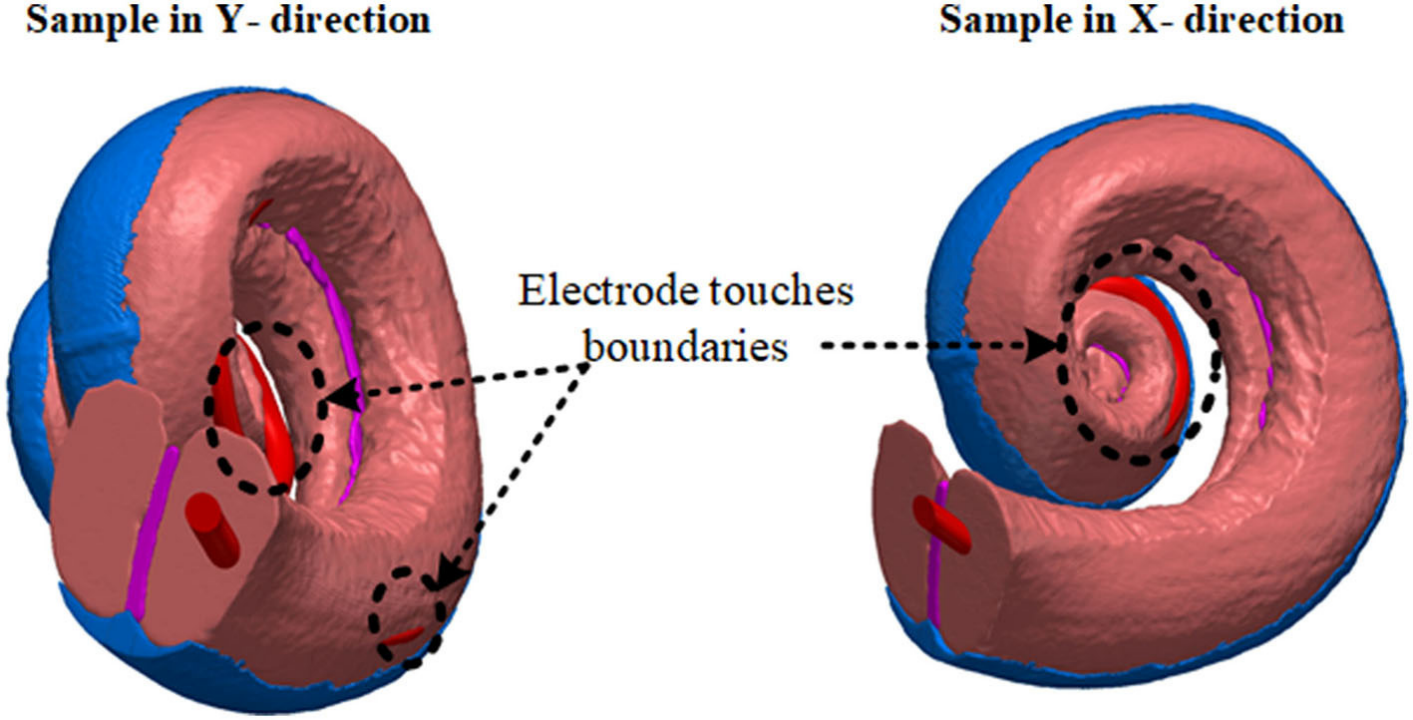
The distance between the anatomical layer and electrode array is not the same for all electrode contacts along the cochlea. This limits the number of samples in the x and y directions. Electrode arrays exit from the scala tympani when they are shifted beyond a certain distance.

First, the electrode array was considered to be placed at the center of the scala tympani. The centerline of the scala tympani was manually generated by calculating the variable cross-section of the scala tympani along with the spiral shape of the cochlea and stored as x, y, and z coordinates in ScanIP software. The electrode array was modeled inside the cochlea by interpolating the center points of the scala tympani and using the sweep function in COMSOL Multiphysics^Ⓡ^ v5.2a (COMSOL, Ltd, Cambridge, UK). Since the electrode's plates are relatively thin, they were designed as boundary surfaces and combined with the electrode array in COMSOL. The 16 electrode array was inserted into the scala tympani at the midscale position. The electrodes were numbered from E1 at the apical end to E16 at the basal end. After placing the 3D model of the electrode in the scala tympani, the electrode model was relocated to evaluate the impact of the proximity to the cochlea wall on the impedance variation. This resulted in six models in the x and six in the y directions as samples shown in Figure 2. Note that the models are equally spaced in both the x and y directions. It was not possible to generate more different electrode array samples due to the shape of the cochlea. The electrode model eventually touched the scala tympani's wall (in both x and y directions) as shown in Figure 3.

In the following subsections, the impact of the electrode array's proximity to the cochlea wall and the depth of the electrode array penetration are investigated based on impedance variations.

#### 2.2.1. Electrode Proximity

In Figure 2A since the cochlea wall is at one surface of the scala tympani, the effect of the proximity to this layer of the electrode impedance in the y direction through the cochlea wall is investigated. The electrode array was shifted in the y direction in incremental steps until the silicone base of the electrode array nearly touched the wall of the scala tympani. The process was repeated in the x direction, Figure 2B, from one outer wall to the opposite outer wall. The step distance between any two models was equal and was defined to obtain significant impedance differences (at least 2%) between adjacent positions. Since the cochlea has a helical shape tapering down from the base to the apex, this limits the generation of more samples due to the electrode array exiting from the scala tympani after a certain distance in both x and y directions as shown in Figure 3. Six different measurements were obtained in each x and y direction. Each electrode position (M1 to M6) was merged in the 3D cochlea volume conductor, discretized and the electrical potential field due to a current input at the electrode was simulated and the impedance was measured.

#### 2.2.2. Electrode Insertion Depth

The simulated 16 electrode array was positioned along the center of the 3D scala tympani. The electrode contacts were designed and combined with the silicone carrier in COMSOL to form an array model where E1 and E16 represent the initial insertion and full insertion depth, respectively. The 3D electrode model was imported into ScanIP to combine with the 3D cochlea model. It was assumed that the electrode array has been inserted in the optimal place (center of the scala tympani) of the cochlea. The electrode array was inserted into the cochlea wall from the apical electrode (E1) until full insertion (E16). The electrical potential distribution was simulated for each electrode insertion and impedance variation was assessed for each electrode accordingly as shown in Figure 8.

### 2.3. Model Validation

Very detailed electrode proximity parametrization based on impedance variation employing the accurate anatomical cochlea model is impractical due to its complexity requiring very long computation times (refer below). Also, it was shown that it was not possible to parameterize the electrode array position within the scala tympani due to the helical shape of the cochlea as shown in Figure 2. The electrode array touched the nearby anatomical layers (as shown in Figure 3), and this limited generation of more samples using an anatomical model. An alternative simplified and sufficiently accurate model of the cochlea and adjacent tissue layers can be represented by geometries (i.e., ellipsoids, cylinders) that describe only the regions of interest (Salkim et al., 2019). This significantly reduces computation times at the cost of some minor added error, allowing practical multiple measurements. Note that the same electrode dimensions and current source were used for all generated models.

The two models were compared based on each electrode impedance variation (as shown in Figure 8) for the full electrode array insertion. The resulting impedance variation with electrode depth was recorded (from E1 to E16) for both anatomical and geometrical models. The resulting error was 1 (minimum) to 2% (maximum) when compared to impedance measurements for the same distance to the cochlea wall as shown in Figure 8. The computation time per measurement for the anatomical model was ~5 h but it was 10 min for the geometrical model. This significantly reduces computation time but still has sufficient accuracy and enables a more detailed parametrization of the proximity of the electrode array based on impedance measurements.

### 2.4. Detailed Electrode Proximity Impedance Parametrization Using a Geometrical Model

**Figure 4:**
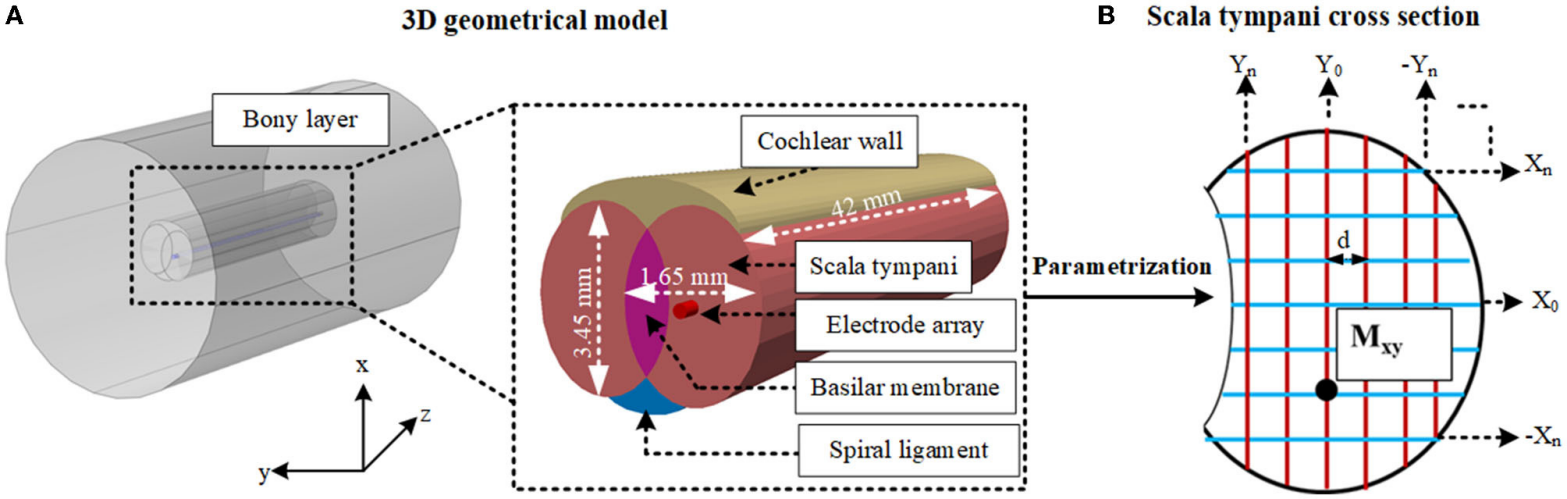
**(A)** Shows the layers of the 3D geometrical model and dimensions of the cochlea. **(B)** Shows the cross-section of the scala tympani and the parametrization of the proximity of the electrode array in this layer. Different samples are highlighted in both directions (X and Y). *M*_*xy*_ shows one of the samples, and d shows the equal distance between each sample for both x and y directions. Other layers are not shown.

**Figure 5:**
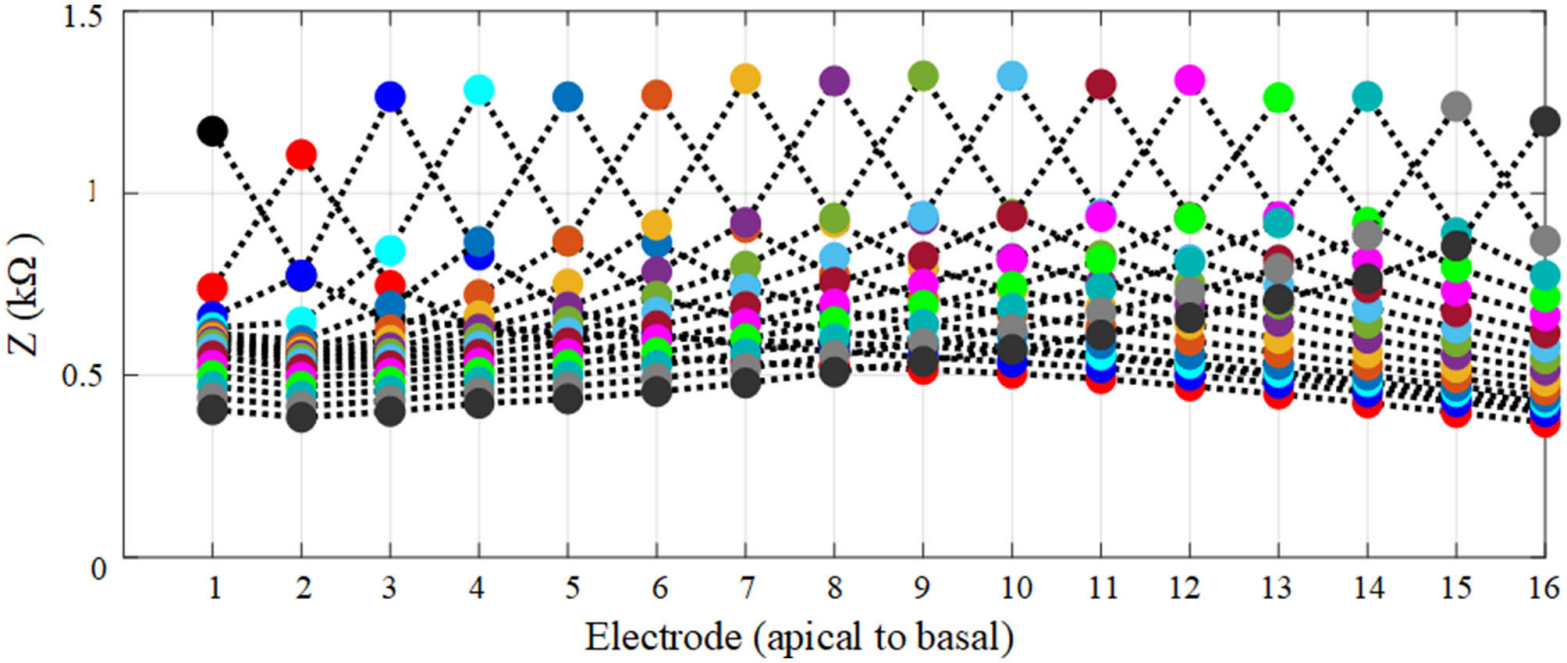
Each spread curve shows impedance variation for each electrode. Impedances were again assessed across all contacts for each activated electrode contact.

To generate the geometrical model the bony layer, scala tympani, vestibular and basilar membrane layer, spiral ligament, and electrode array were constructed based on ellipsoids and cylinders as shown in Figure 4A in COMSOL with relatively larger element dimensions compared to the anatomical model. The stria vascularis layer was modeled as “contact impedance” during simulations. The electrode array was initially inserted into the scala tympani and impedance variation was measured to assess the difference between the initial and full insertion of the electrode. Each electrode, in turn, was activated, and impedance was calculated for electrode contacts. As shown in Figure 5 the impedance varies for each electrode contact in agreement with (Vanpoucke et al., 2011). The parametrization process was assessed based on the full insertion of the electrode within the scala tympani. Measurements of electrode impedances were sequentially made between each contact and the ground ([E1 to the ground], [E1 to the ground, E2 to the ground], … [E1 to ground,…E16 to ground]). For each electrode insertion from E1 to E16, impedances were again assessed across those contacts that were already in the scala tympani. This resulted in 136 impedance recordings for specified proximities to the cochlea wall (e.g., *X*_*n*_, *Y*_0_) until a certain distance approached the tympani border (e.g., *X*_0_). The measurements were limited to these areas to reduce computation costs.

**Figure 6:**
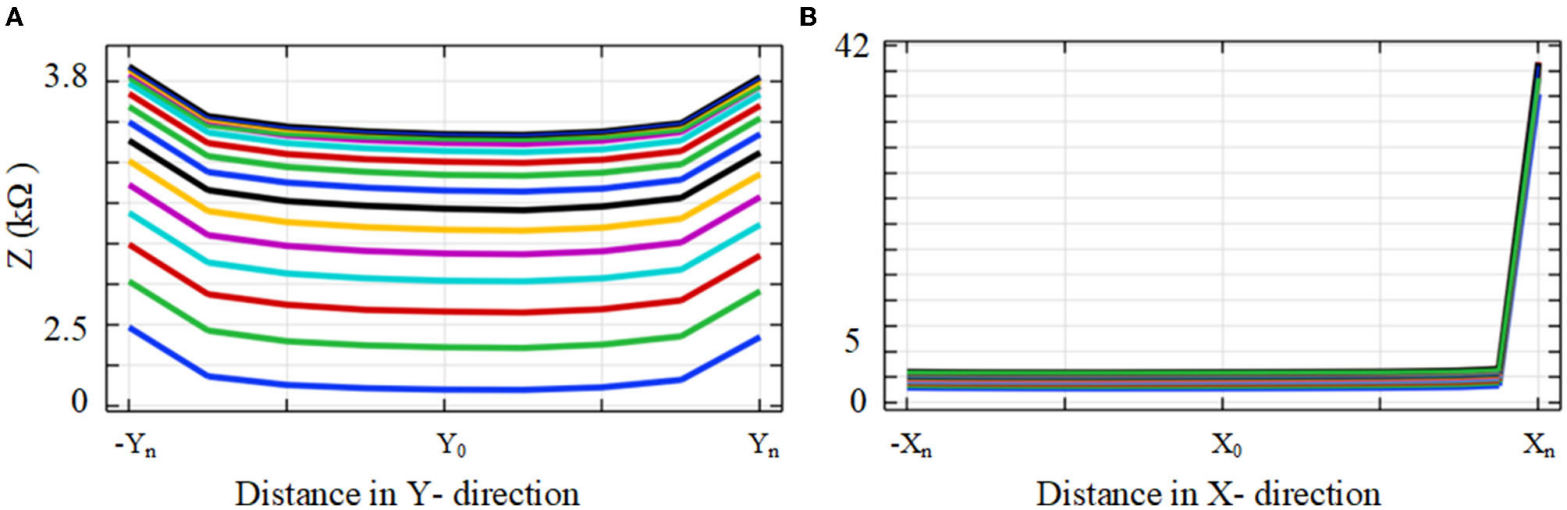
**(A)** Electrode array proximity was parameterized for the scala tympani border in the y direction and **(B)** cochlea wall in the x direction. *Y*_0_ was kept constant and electrode proximity to the cochlea wall in the x direction was parameterized. The same process was repeated for the y direction.

The electrode position parametrization was based on an 80 × 60 matrix of cross points overlaying the oval-shaped cross-section shown in Figure 4B, resulting in somewhat fewer than 144 positions by ignoring insignificant variation in impedances (as shown in Figure 6). A reduction in computation time was made by selecting samples in X and Y positions (Figure 4B). First, a sample in the x direction (e.g., *X*_0_) was kept constant and the electrode position was swept up to 12 increments in the y direction to very close to the tympani border or basilar membrane. This process was repeated for the remaining samples (from *X*_0_ to *X*_*n*_). The same procedure was repeated with X and Y interchanged for −*Y*_*n*_ to *Y*_*n*_. At each point, the electrode electrical potential was simulated and its impedance to the ground was recorded. Note that the impedance was not calculated for the models that touched the cochlea wall or border of any adjacent layers. This provided sufficient detail for analysis. Since the variation when approaching the cochlea wall is vital, the impedance variation was calculated for all electrode contacts in the x direction but it was recorded for E1, E6, E11, and E16 in the y direction as discussed in Section 3.

### 2.5. Tissue Conductivity and Boundary Conditions

Once the anatomical cochlea volume conductor model and electrode array settings were completed, the electrical characteristics for each tissue layer were assigned using the parameters in Table 1 to perform the impedance measurements. The simulations were solved based on Dirichlet boundary conditions using (1) which approximates to ground at the infinity boundary condition.


(1) $$\begin{array}{r} V\left(\delta \Omega \right)=0 \end{array}$$

where *V* shows the electrical potential and δΩ represents the outermost surface layer of the model. The conductivities of tissue layers within the volume conductor are listed in Table 1. The electrical features of the cochlea layers have been reported in numerous studies (Frijns et al., 1995; Hanekom and Hanekom, 2016), and the conductivity values that are currently used in the computational modeling of the cochlea used in this study are shown in Table 1. They are assumed to be isotropic as there is no data in the literature on the anisotropy of the layer conductivities of the cochlea except for the bone layer. The quasi-static approximation was used as detailed in the following subsection. The conductivities of the scala tympani and scala vestibuli were assumed to be the same since both layers are composed of the same fluid (perilymph) and possess similar electrical characteristics. The thin anatomical layers around the scala (veins, nerve trunk) were not considered in the final volume conductor model; it was assumed they have a negligible effect on impedance when typical CI current pulses are applied. The external surface of the membrane was insulated to prohibit current flow from the scala tympani into the non-conductive middle ear air space. Finally, the surface electrode remaining external to the scala tympani was grounded to represent the current sink.

### 2.6. Computing the Electrical Potentials in a Volume Conductor

Each model was imported into COMSOL for finite element analysis. Models were then discretized using tetrahedral finite elements for numerical solutions of partial differential equations in COMSOL. Each simulation was solved iteratively on a 64-bit multicore processor using the conjugate gradients method. The accuracy of the simulation is proportional to the volume conductor mesh resolution. The scalas and the tissue layers near the scalas were meshed using a minimum element size of 1 μm and a relatively lower growth rate (1.1) and the remaining tissue layers were meshed with relatively larger minimum element sizes (e.g., 0. 1–1 mm) to obtain sufficient accuracy while reducing excessive computation time. Mesh settings for the electrode (electrodes) were adaptively adjusted to different sizes and growth rates in different models. Since the outermost layer (bone) was far from the region of interest, the discretization element size was selected to be larger (i.e., known as normal tetrahedral setting) than the cochlea layers. The number of elements varied approximately between three and five million during the discretization process, depending on the model.

In this study, simulations calculated the electrical potential distribution within the volume conductor using the quasi-static approximation of the Laplace equation:


(2) $$\begin{array}{r} \nabla \cdot \left(\sigma \nabla V\right)=0 \end{array}$$

where σ is the tissue conductivity (as shown in Table 1), and *V* is the electrical potential in the representative geometry. The electrical potential variation for each model was simulated by applying a 34 μA current to calculate impedance measurements as shown in (3). The impedance *Z*_*el*_ to ground for each model was derived from (3)


(3) $$\begin{array}{r} Z_{el}=V_{el}/I_{el} \end{array}$$

where *V*_*el*_ is the resulting electrode potential, and *I*_*el*_ is the applied quasi-static current (chosen to be unity). Since the study is based on quasi-static approximation due to the lack of the dielectric parameters of the cochlea, the electrode-tissue interface contact impedance was assumed to be zero. The appropriate continuity conditions were implemented at the boundary of the different domains to provide a unique solution.

## 3. Results

In this section, impedance variation was initially analyzed based on the anatomical cochlea model. After comparing the results for both the anatomical and geometrical models, parametrization results were generated based on the geometrical model.

### 3.1. Impedance Measurements in the Anatomical Model

**Figure 7:**
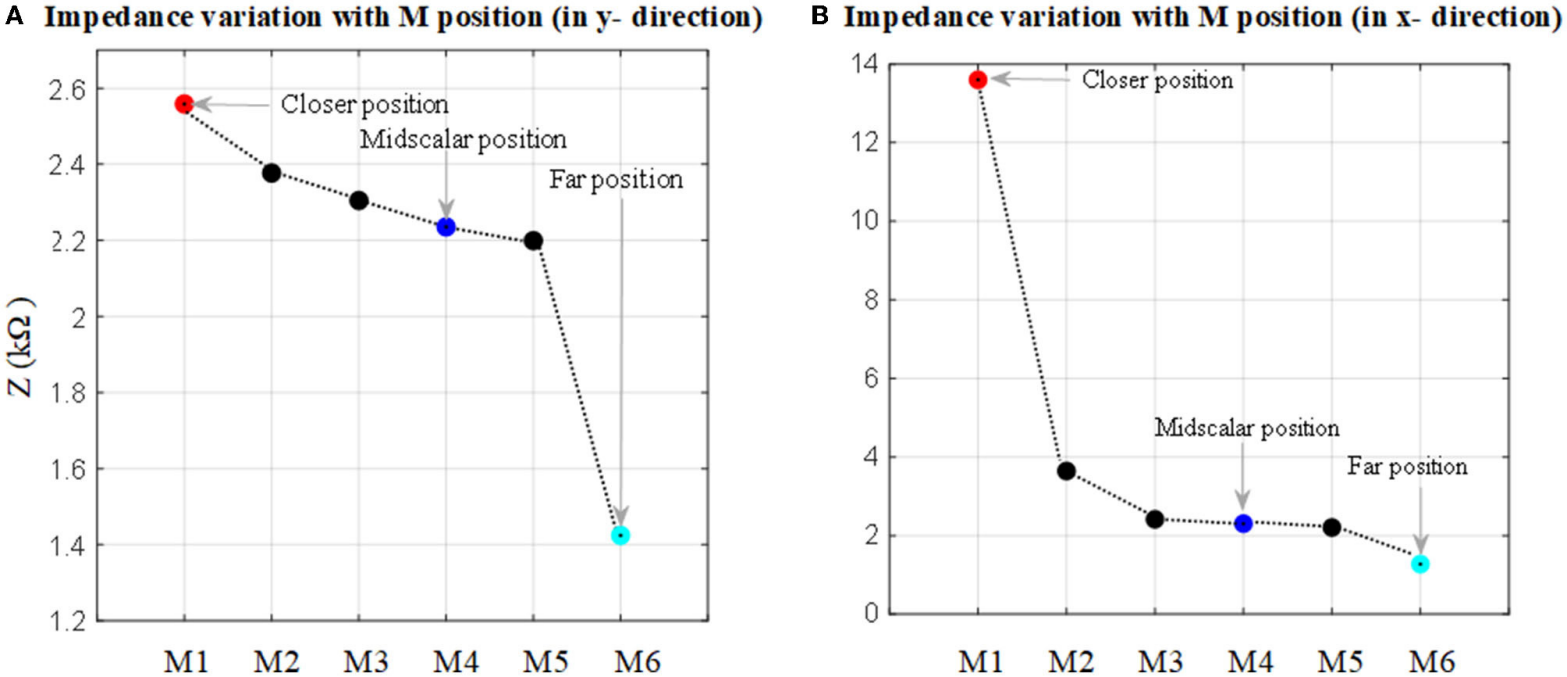
Impedance to ground variation with the position in the cochlea for an electrode when the 16 electrode array is fully inserted. **(A)** Electrode impedance variation with proximity to the basilar layer in the y direction (refer to Figure 2A). **(B)** Electrode impedance variation with proximity to the cochlea wall in the x direction (refer to Figure 2B).

The impedance to ground variations of the electrode (of the fully inserted array) with electrode proximity to the cochlea wall for x and y directions is shown in Figure 7. Each measurement was color-coded and labeled with the proximity to the cochlea wall or border of the nearby tissues. For the x direction a red circle indicates its relatively closer position to the cochlea wall but not touching, a blue circle indicates a mid-scalar position and a cyan circle is when the electrode is at the furthest position from the cochlea wall relatively close to (but not touching) the outer wall of scala tympani; similarly for the y direction.

As shown in Figure 7A, there is a direct relationship between impedance magnitude and electrode proximity to the scala tympani border in the y direction. The impedance changes increase with the electrode proximity to the basilar membrane. They increased by about 12% when the closer position of the electrode is compared to the mid-scalar one. There is a notable change in impedance magnitude when the electrode is moved away from the sensitive layer (basilar membrane). The impedance varies from 2.6 to 1.4 kΩ. Figure 7B shows the results for the electrode proximity to the cochlea wall in the x direction. There is significant variability in the impedance when the electrode is placed closer to the cochlea wall, compared to the mid-scalar and furthest position. The impedance varies from 2 to 13.5 kΩ.

### 3.2. Model Validation

**Figure 8:**
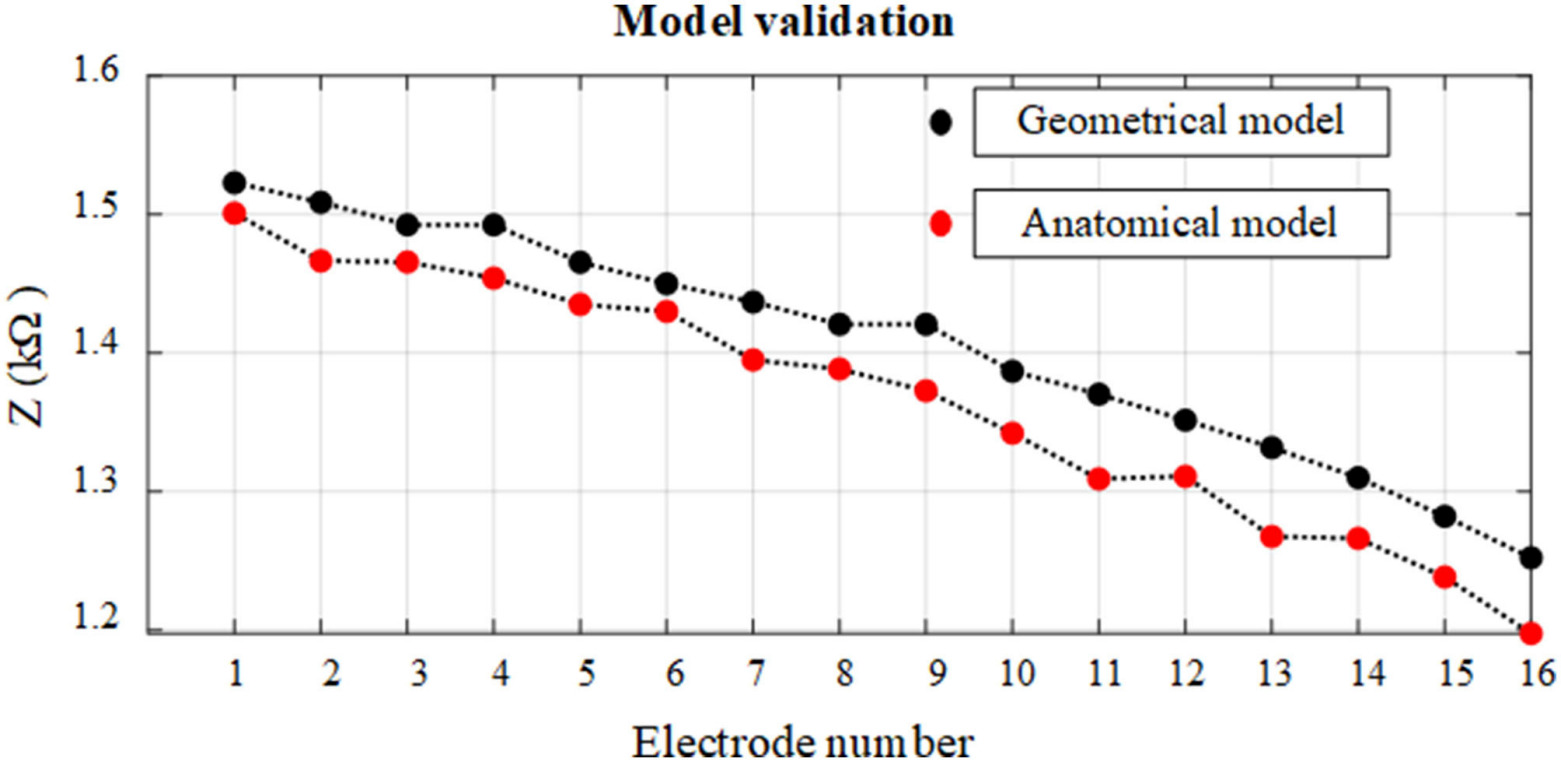
Relationship between impedance variation and electrode insertion depth for anatomical and geometrical cochlea models. The electrodes on the fully inserted array are numbered from E1 to E16.

The electrode array is fully inserted into the cochlea and is assumed to be at the mid-scalar position. The anatomical and geometrical models were compared based on electrode insertion from the apex (E1) to basal (E16) electrode impedance variation. The impedance variation between these models based on a fully inserted electrode array is highlighted in different colors and shown in Figure 8. The impedance of electrodes at different insertion depths shows an approximately linear increasing change with electrode depth for both models. The impedance measurement is slightly higher using the geometrical model compared to the anatomical model. The impedance difference between the two models for different electrodes varies between 1 and 2%, providing sufficient accuracy with far less computation cost.

### 3.3. Measurements of Impedance Variation With Electrode Proximity Based on a Geometrical Model

**Figure 9:**
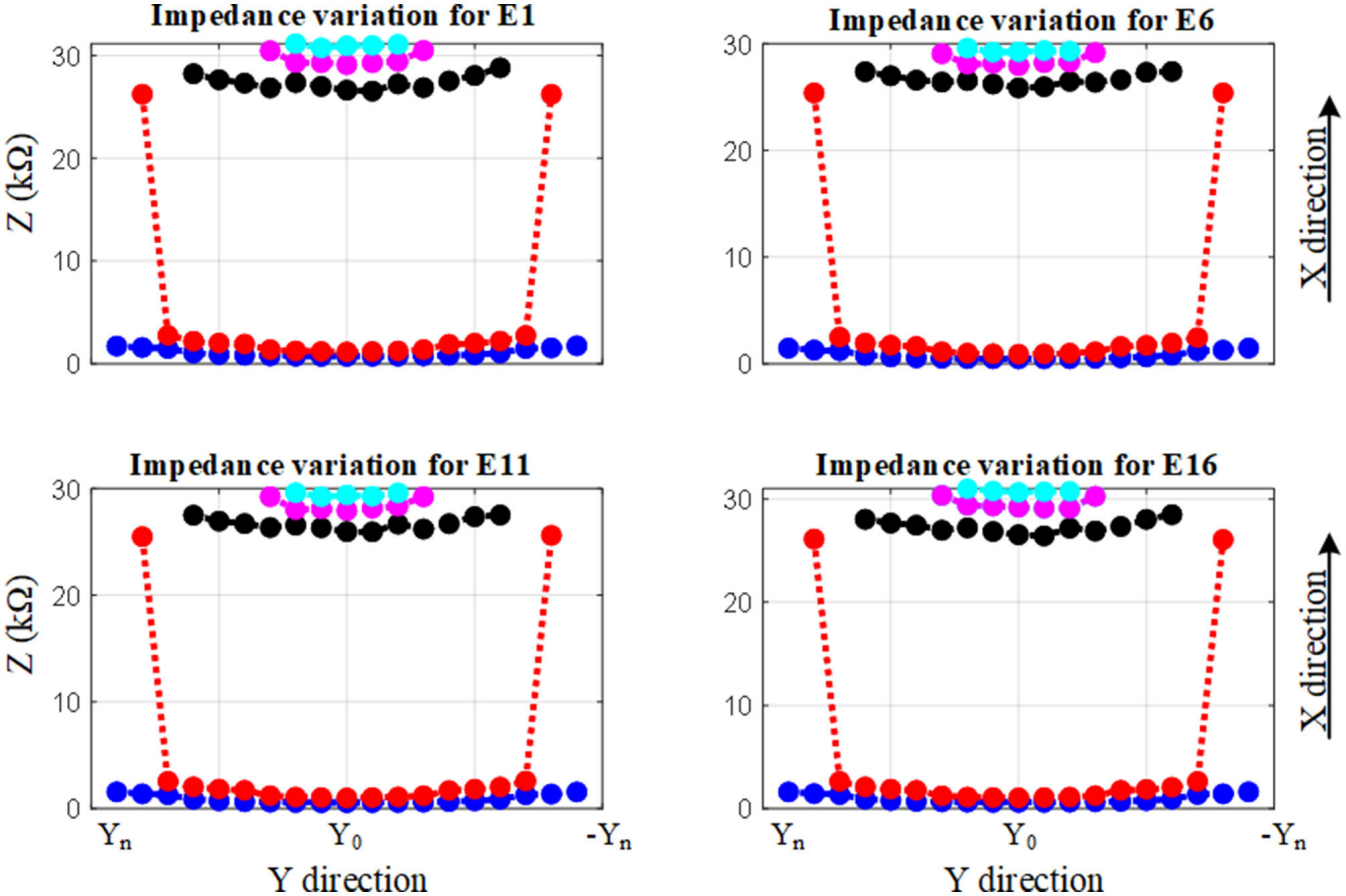
Impedance variation for different electrode proximities to the basilar membrane and scala tympani. The electrode was swept in the y direction for each sample in the x direction.

**Figure 10:**
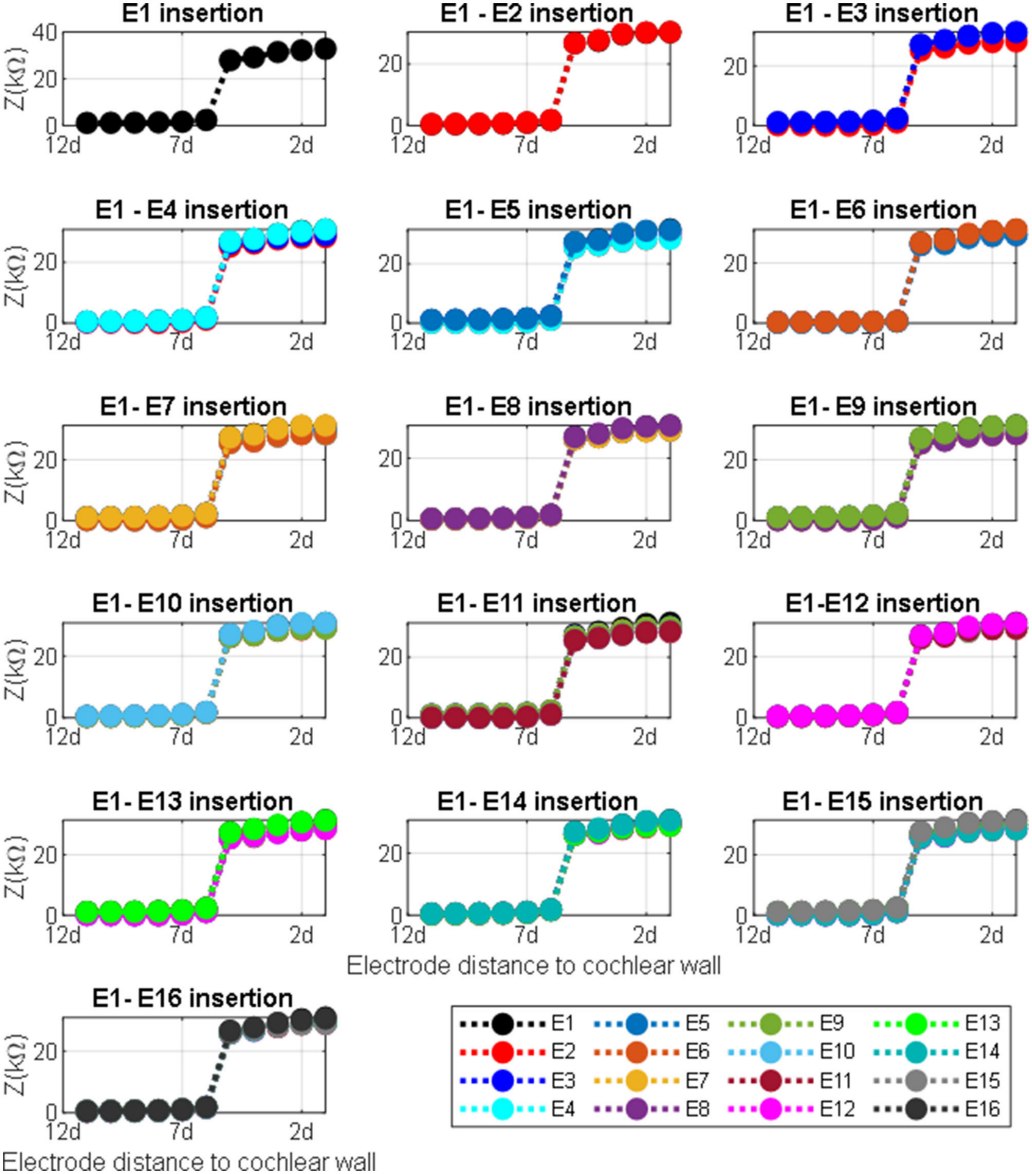
Impedance variation for different electrode proximities to the cochlea wall. The electrode was swept in the x direction for each sample in the y direction for defined electrode insertion. Distance d = proximity.

The impedance variation with different proximities of the electrode to the cochlea wall in the y and the x directions are shown in Figures 9, 10, respectively.

Figure 9 shows the impedance variation for the same samples of the electrode contacts such as E1, E6, E11, and E16. Examination of Figure 9 shows that there is a correlation between the electrode impedance and its distance to the basilar membrane or scala tympani outer wall for all positions. It shows that the magnitude of the impedance increases when the electrode is closer to these boundary layers for all samples. In particular, the results highlighted in red indicate that the magnitude of the impedance is considerably changed in the y direction for a certain sample in the x direction. As the electrode array is placed closer to the cochlea wall (distance in the x direction), this resulted in the highest magnitude difference in impedance which is in agreement with Figure 10. On the other hand, the results for the electrode array that is placed toward the center of the scala tympani show the lowest impedance difference for different distances.

Figure 10 shows impedance variation for sequential electrode insertion (E1 insertion, E1 to E2 insertion… E1 to E16 insertion) for different proximities (d to 12d) of the cochlea wall. As shown in the subplots in Figure 10, there is a relationship between the magnitude impedance and distance in the x direction. In particular, there is a significant impedance variation when the electrode array is placed at a certain distance to the cochlea wall (5d) for all electrode contacts compared to the other distances. The magnitude of the impedance is approximately increased from 2 to 28 kΩ for all electrode insertion samples. Although the magnitude of the impedance is increased with closer proximity (<5d), this is not significant. The remaining distances (from 12d to 6d) do not show notable variation in impedance being relatively far from the cochlea wall.

## 4. Discussion

One of the key requirements of the CI is the positioning, or geometry, of the electrode array relative to cochlea anatomy (Finley and Skinner, 2008). The experimental visual inspection of the implant is limited (Kratchman et al., 2016). Bio-modeling is increasingly becoming an alternative option to the design and optimization of biomedical devices (Hanekom and Hanekom, 2016; Salkim et al., 2019). Specifically, the electrode array positioning within the anatomical layer can be readily investigated using these models. In such models, the electrical potential is simulated within the volume conductor using appropriate boundary conditions in relation to the associated tissue and electrode electrical parameters.

In this article, a detailed 3D anatomical model of the human cochlea was generated using an individual image dataset. Different set models of the electrode array were generated based on a commercial electrode array and each model was merged with a 3D model of the cochlea to examine the impact of the electrode proximity to the cochlea wall. Using a detailed anatomical model may not be an optimal method to accomplish such an investigation due to its computation cost and the limitation of the model samples. As an alternative, a 3D geometrical model was constructed based on the anatomical model to readily parameterize the proximity of the electrode. Thus, the impact of electrode proximity to the cochlea wall and the electrode insertion depth based on impedance measurements were examined to investigate whether the impedance variation can be a guide of the electrode positioning during surgery. Different electrode array models, from far to close to the cochlea wall, within the 3D cochlea were developed.

The results showed that the impedance varied with both proximity and insertion depth as shown in Figures 7–10. As shown in Table 2, these results are in line with other clinical real-time and computational measurements (Tan et al., 2013; Giardina et al., 2017; Pile et al., 2017). The results for the anatomical model (Figure 7) showed that there was a significant impedance increase (350%, comparing M1-M2 to M2-M3) when the electrode was placed closer to the cochlea wall in the x direction. This may be due to the lower conductivity of the cochlea wall which is much lower than the center of the scala tympani. Since the 3D model of the basilar membrane was not exactly perpendicular to the y-axis, M1 is closer to the basilar membrane compared to M6 in the y direction. This leads to the observed higher impedance variation in M1 when compared to the remaining ones in the y direction. This may be due to the basilar membrane resistivity which is much higher than the center of the scala tympani (Frijns et al., 1995). The strong impedance dependence of the electrode proximity near the cochlea wall is a useful characteristic.

**Table 2:** Comparison of this study and published results on the electrode impedance variation range.

**Study**	**Impedance range (kΩ)**	**Electrode**	**References**
Experimental	5–22	Contour advance	Tan et al., 2013
Experimental	3–25	Contour advance	Pile et al., 2017
Experimental	2–8	Flex	Bruns et al., 2021
Modeling (Computational)	2–5	Flex	Bruns et al., 2021
Modeling (Phantom)	1–25	HiFocus^*TM*^ SlimJ	Giardina et al., 2017
Modeling (Computational)	3–4.5	HiFocus^*TM*^ SlimJ	Salkim et al., 2020
Modeling (Computational)	1–38	HiFocus^*TM*^ SlimJ	This work

The impedance variation of electrode insertion depth was compared based on anatomical and geometrical models to examine the use of the geometrical model for the further assessment of the impedance variation as shown in Figure 8. The results showed that the geometrical model can be used to parameterize electrode array in the scala tympani with a maximum error of 2%.

The results based on geometrical model simulations in Figures 9, 10 for different points in the x and y directions in the scala tympani indicated that the variation in impedance can be correlated with the proximity of the electrode array to the cochlea wall in agreement with the clinical results (Tan et al., 2013; Pile et al., 2017). This difference can be readily observed in the impedance variation when the electrode array was placed closer to the cochlea wall in the x direction and basilar membrane in the y direction. Although there is no significant change in impedance when the electrode array is swept in the y direction for a certain distance in the x direction, a notable variation was observed for a larger distance in the x direction as shown in Figure 9. It is noted that impedance increases when the electrode array is shifted toward the inner and outer scala tympani as shown in Figures 6, 9. This may be a guide the surgeon to safely place the electrode in the scala tympani without touching the borders in the y direction. The same variation trend was partially observed for anatomical model results in Figure 7A. This is due to the generation of the limited samples in the y direction.

There was a considerable change in the magnitude of the impedance for all insertions based on different proximities to the cochlea wall in the x direction as shown in Figure 10 subplots. The impedance increased by 1,400% when the electrode was moved from 6d to 5d (relative distance). Note that there was a small impedance increment when the electrode was placed further away from the cochlea wall in the x direction. It has been shown that the majority of CI current is confined to the scala timpani due to a relatively higher current pathway compared to the transversal current pathways toward the cochlea wall (Vanpoucke et al., 2004). As the current is limited to the scala tympani, the return path to the ground becomes longer and the cochlea conductive space becomes narrower as the electrode array is inserted deeper into the scala tympani. This may explain why the total impedance increases with both insertion depth and proximity to the cochlea wall (Tan et al., 2013; Pile et al., 2017), consistent with the results in this study. Thus, the electrode array proximity sample (relative distance 6d) is much more sensitive and specific to detecting which electrodes are in very close proximity to the cochlea wall. Each model provides discrete but complementary information regarding the position of the electrode relative to the cochlea wall or the borders of the scala tympani, which may be clinically valuable in assessing the electrode positioning. In this way, the surgeon could adjust the position of the electrode in the scala tympani during the insertion process if the results showed around this threshold.

The conductivity values that are most commonly used in current computational modeling were used in this study. The impact of the conductivity variation on the impedance variation was investigated. Since the most important layer is scala tympani for the electrode insertion guidance, the conductivity of this layer was changed in ±5% steps and the resulting simulated electric potential was recorded to investigate the impact of the conductivity on the impedance variation. It was shown that there is no significant change in impedance variation (error < 1%) for ±10% variation in the conductivity.

Although various impedance values were recorded when the electrode array was placed relatively close to the cochlea wall, it has been shown in modeling and experimental studies that there is a significant increment in the impedance variation when the electrode array is placed very close to the cochlea wall. This may help to alert the surgeon to further action.

A limitation of this study is the assumption that all tissue layers are purely conductive and isotropic without considering dielectric properties. Also, it was assumed that each contact of the electrode array has equal proximity to the cochlea wall for each design.

The results of this study demonstrate that impedance variation can be a guidance marker for the positioning of the electrode array. The method could be used to develop a real-time guidance tool for the surgeon to prevent hearing loss by avoiding the electrode array touching the cochlea wall and delicate tissue layers (e.g., basilar membrane, hair cells) during insertion.

## 5. Conclusion

Accurate anatomical and geometrical volume conductor models of a human cochlea provide useful tools for studying the relationship between electrode impedance and electrode position in the scala tympani. Using the geometrical model of the cochlea and combined with adequate electrical parameters of CI, the parametrization processes were applied to construct an impedance variation map based on both electrode array insertion depth and electrode proximity to the anatomical layers at the vicinity (e.g., cochlea wall). The method has been shown to identify the impedance variation levels for the electrode proximity position and electrode insertion. The results of this study suggest it may be clinically applicable and lead to optimal electrode array positioning if they are validated with the experimental study. Future study will involve an experimental study of the electrode array positioning in temporal bone and cadaveric tests to further validate the relationship between impedance and electrode position and compare it with computational results.

## Data Availability Statement

The raw data supporting the conclusions of this article will be made available by the authors, without undue reservation.

## Author Contributions

ES designed the models, performed the simulations, analyzed the data, and wrote the manuscript. MZ, DJ, and SS reviewed the manuscript and contributed to the research. AD supervised the research and reviewed and edited the manuscript. All authors approved the final manuscript.

## Funding

This study was supported by the Engineering and Physical Sciences Research Council under grant EP/R511638/1.

## Conflict of Interest

The authors declare that the research was conducted in the absence of any commercial or financial relationships that could be construed as a potential conflict of interest.

## Publisher's Note

All claims expressed in this article are solely those of the authors and do not necessarily represent those of their affiliated organizations, or those of the publisher, the editors and the reviewers. Any product that may be evaluated in this article, or claim that may be made by its manufacturer, is not guaranteed or endorsed by the publisher.
